# Seasonal Changes in the Elemental Composition of Five Valuable Fish Species (*Sparidae*) from Bozcaada, North Aegean Sea: A Health Risk and Nutritional Benefit Assessment

**DOI:** 10.3390/foods14020324

**Published:** 2025-01-20

**Authors:** İlknur Yuvka, Ali Rıza Kosker, Mustafa Durmus, Yılmaz Ucar, Yesim Ozogul

**Affiliations:** 1 Department of Seafood Processing Technology, Faculty of Fisheries, Cukurova University, Adana 01330, Balcali, Türkiye; ilknuryuvka@gmail.com (İ.Y.); akosker@cu.edu.tr (A.R.K.); mdurmus@cu.edu.tr (M.D.); 2 Vocational School of Aladag, Department of Forestry, Cukurova University, Aladag, Adana 01330, Balcali, Türkiye; yucar@cu.edu.tr

**Keywords:** seafood safety, essential minerals, toxic metals, health risk assessment

## Abstract

This study investigates the seasonal variations in the elemental composition of five economically valuable *Sparidae* fish species from Bozcaada, North Aegean: red seabream (*Pagrus major*), gilthead seabream (*Sparus aurata*), saddled seabream (*Oblada melanura*), white seabream (*Diplodus sargus*), and common dentex (*Dentex dentex*), with a focus on both essential minerals and toxic metals. Fish samples (*n* = 10 per species per season) were collected across four seasons, and their weights and lengths were recorded. The concentrations of elements such as calcium, potassium, magnesium, phosphorus, copper, iron, manganese, zinc, chromium, nickel, selenium, cadmium, and mercury were analyzed using Inductively Coupled Plasma Mass Spectrometry (ICP-MS). The elemental concentrations varied as follows: Ca (11,388.46–55,470.76), K (17,230.83–27,594.86), Mg (1436.02–2326.73), Na (1962.30–7847.41), P (13,112.11–15,516.57), Fe (107.61–282.00), Cu (36.44–59.13), Mn (6.19–19.87), Zn (98.67–256.26), Cr (4.54–11.96), Ni (6.33–13.89), Se (0.82–7.33), Cd (0.08–0.32), and Hg (0.08–1.50) mg/kg. Health risk assessments, including Estimated Weekly Intake (EWI), Target Hazard Quotient (THQ), and Cancer Risk (CR), were calculated for both adult and child consumers. The results showed that while the essential minerals remained within safe limits, seasonal variations in the concentrations of toxic metals could pose potential health risks, particularly with frequent consumption. This research provides valuable insights into balancing the nutritional benefits and safety of fish from Bozcaada, offering recommendations for informed consumption and public health policies aimed at optimizing benefits while minimizing risks.

## 1. Introduction

Seafood is an essential component of the human diet, offering high-quality protein, essential vitamins, and minerals. It is renowned for its numerous health benefits, including promoting cardivascular health, supporting cognitive development, and providing anti-inflammatory effects [1,2,3,4]. Despite these advantages, seafood can also present risks due to the potential exposure to harmful elements such as mercury, cadmium, and arsenic. These toxic metals may pose significant health threats such as gastrointestinal and kidney dysfunction, nervous system disorders, skin lesions, vascular damage, immune system dysfunction, birth defects, and cancer if consumed in excessive amounts over time [5,6,7,8].

In recent years, concerns about the nutritional value and safety of seafood have led to increased research into its biochemical composition and the concentrations of trace elements in various species [9,10,11,12,13]. However, the determination of metal content in seafood alone may be insufficient to identify potential benefits and risks for consumers. Therefore, risk assessment parameters such as Estimated Weekly Intake (EWI), Target Hazard Quotient (THQ) and Lifetime Cancer Risk (CR) are widely used to assess the safety of seafood [14,15,16,17,18]. These frameworks help determine safe consumption limits due to metal intake based on frequency of consumption and guide recommendations to minimize health risks. Therefore, monitoring the metal content in marine products and calculating possible health risks is important for consumers’ access to healthy food [9,12,19]. Moreover, metal levels in seafood and risk assessments based on these levels will contribute to filling the gap in the literature and increasing the awareness of consumers.

The Northeastern Mediterranean, namely the Aegean Sea region, is also an area affected by the Marmara and Black Sea maritime transportation routes. The Aegean region of Türkiye, including Bozcaada, is recognized for its rich marine biodiversity and the high quality of its seafood, which supports both local consumption and commercial fisheries [20,21]. Bozcaada is a Turkish island in the northeastern Aegean Sea, south of the Dardanelles Strait, and is an area where regular pollution monitoring activities are required due to its location. Because of the intense maritime traffic in the Turkish Straits and the ecological situation in the Black Sea, there are two types of potential pollution in the Northern Aegean Sea. The first one is related to the increasing number of accidents in the Turkish straits and the inevitable pollution caused by them [22]. The second is related to brackish waters and pollutant discharges entering the North Aegean Sea from the Black Sea through the Çanakkale strait. It shows that the long-term distribution and accumulation of pollutants of Black Sea origin, which are continuously discharged into the North Aegean from the Dardanelles outlet, are very high in the coastal waters of the islands of Thassos, Samothrace and Limnos, and in the mainland coastal waters between Alexandroupoli and the Gulf of Strymonikos, during the summer and autumn months when strong water column stratification occurs [23]. On the other hand, long-range transport of air pollutants from industrial and urban environments from Eurasia to the far periphery of the Northeastern Mediterranean, i.e., the North Aegean Sea region, during the summer months when synoptic Etesian wind conditions prevail, can significantly affect the quality of air in remote areas [24].

This study focuses on five key fish species—red seabream (*Pagrus major*), gilthead seabream (*Sparus aurata*), saddled seabream (*Oblada melanura*), white seabream (*Diplodus sargus*), and common dentex (*Dentex dentex*) from Bozcaada. These species are not only of economic importance but are also widely consumed by locals and tourists, contributing to the region’s dietary diversity [25,26]. In this context, the present study aimed to evaluate the nutritional profile and health risks associated with the consumption of five economically valuable Sparidae species from Bozcaada. By focusing on both essential minerals and potentially toxic metals, this research provides a comprehensive assessment of seasonal variations in their elemental composition. Health risk evaluations for both adults and children were conducted across different consumption frequencies (1, 3, and 5 days per week) to better understand the potential risks.

## 2. Materials and Methods

### 2.1. Fish Collection and Identification

Sampling for the five fish species (Table 1) was conducted by trawling between coordinates 39°53′07.4″ N, 26°00′18.7″ E and 39°53′42.5″ N, 26°06′47.4″ E in Bozcaada, northeastern Aegean region. For each species, 10 samples were selected for each season. Sampling was performed over four seasons (winter, spring, summer, and autumn) between December 2019 and November 2020.

### 2.2. Elemental Analyses

The determination of metal concentrations in the fish samples was conducted following a modified version of the Canlı and Atlı method [27]. Approximately 0.5 g of wet fish tissue was digested using a combination of 4 mL concentrated nitric acid and 2 mL perchloric acid (both obtained from Merck, Darmstadt, Germany). The samples were then heated on a hotplate (Velp-Scientifica, Usmate Velate, Italy) at a temperature of 150 °C until complete dissolution of the tissue was achieved. The concentrations of macro elements (such as Na, Mg, P, K, and Ca), trace elements (including Fe, Cu, Mn, Zn, Cr, Ni, and Se), and toxic metals (Cd and Hg) were quantified in milligrams per kilogram (mg/kg).

The elemental analysis was performed using an Inductively Coupled Plasma Mass Spectrometer (ICP-MS), model Agilent 7500ce (Agilent Technologies, Tokyo, Japan). The analysis was conducted at Çukurova University Central Research Laboratory (CUMERLAB). Each sample was analyzed in triplicate to ensure accuracy. The operating parameters for ICP-MS were as follows: radio frequency power of 1500 W, plasma gas flow rate of 15 L/min, auxiliary gas flow rate of 1 L/min, carrier gas flow rate of 1.1 L/min, spray chamber temperature maintained at 2 °C, sample depth of 8.6 mm, sample introduction rate of 1 mL/min, and a nebulizer pump speed of 0.1 rps. For calibration purposes, a High-Purity Multi-Standard solution (HPS, Charleston, SC, USA) was employed. Calibration curves were prepared through serial dilutions of stock solutions containing the target elements. Calibration standards for toxic metals were prepared within the range of 1–50 ppb (0.001–0.050 mg/L), while for macro and trace elements, they were in the range of 1–50 ppm (1–50 mg/L).

### 2.3. Health Risk Estimation

To evaluate the potential health risks associated with consuming economically important fish species from the northeastern Aegean region, three risk indicators—Estimated Weekly Intake (EWI), Target Hazard Quotient (THQ), and Cancer Risk (CR)—were calculated. These metrics were computed considering different consumption frequencies: once, three times, and five times per week. The risk assessments were performed separately for adults and children, using body weight assumptions based on U.S. Environmental Protection Agency (EPA) guidelines [28]. For adults, an average body weight of 70 kg and a lifetime duration of 70 years were assumed, while for children, the assumed body weight was 32 kg and the exposure duration was set at 7 years [29].

The EWI was calculated using the following formula:

*EWI = (C_M_* × *IR)/BW* (1)

In this equation, CM refers to the average annual concentration of the metal in muscle tissue (mg/kg), IR is the intake rate (based on consumption frequencies of 1, 3, or 7 times per week), and BW represents the body weight of the consumer (70 kg for adults and 32 kg for children). The intake rates were obtained from FAOSTAT data, which indicated that the average daily fish consumption in Turkey is 18.36 g per person. Weekly consumption levels were extrapolated accordingly to match the different consumption frequencies considered in the study. The obtained EWI values were compared against the Provisional Tolerable Weekly Intake (PTWI) limits set by the World Health Organization (WHO) and the European Food Safety Authority (EFSA). THQ (Target Hazard Quotient) is the expression of the ratio between the reference dose (RfD) of metals and the exposure rate. THQ is used to express the non-carcinogenic risks of metals.

The THQ, used to assess non-carcinogenic risks, was calculated using the U.S. EPA method [30]:

*THQ = [(EF* × *ED* × *IR* × *C_M_)/(RfD* × *BW* × *AT)]* × 10^−3^ (2)

where EF is the exposure frequency, set at 52, 156, and 365 days per year for consumption frequencies of once, three times, and seven times a week, respectively. ED is the exposure duration (70 years for adults, 7 years for children), IR is the consumption amount, CM is the annual average metal concentration in the tissue, RfD is the reference dose for the respective metals, and AT is the averaging time (non-carcinogenic averaging period, calculated as ED × 365 days). If the calculated THQ value exceeds 1, it suggests a potential risk of non-carcinogenic health effects.

Cancer Risk (CR) calculations were performed based on the U.S. EPA method [30]: to estimate the lifetime probability of developing cancer due to metal exposure through fish consumption.

*CR = [(EF* × *ED* × *IR* × *C_M_* × *CsF)/(BW* × *AT)]* × 10^−3^ (3)

where CsF is the Cancer Slope Factor, which indicates the carcinogenic potency of a metal. The CsF values for Cr and Cd were used as 0.5 and 6.3, respectively. A CR value greater than 10^−5^ indicates a significant risk of cancer [30].

### 2.4. Statistical Analyses

All measurements were taken in triplicate, and the results are presented as mean values with their corresponding standard deviations. Normality of the data was assessed using the Shapiro–Wilk test. One-way Analysis of Variance (ANOVA) was applied to identify statistically significant differences among the groups, and Duncan’s multiple range test was conducted to pinpoint these differences at a significance level of *p* < 0.05. Statistical analyses were performed using SPSS software, version 22 (SPSS Inc., Chicago, IL, USA).

## 3. Results and Discussion

### 3.1. Metal/Metalloid Levels of Fish Samples

#### 3.1.1. Macro Elements

The macro element levels of Ca, K, Na, Mg, and P were measured in five fish species (Table 2). The highest Ca value was observed in *P. major* (55,470.76 ± 1277.40 mg/kg) sampled in winter and the lowest value was observed in *D. dentex* (11,388.46 ± 651.80 mg/kg) sampled in spring. The highest Ca value was observed in *P. major* sampled in winter and spring (55,470.76 ± 1277.40 and 52,357.53 ± 2927.60 mg/kg) and in *D. dentex* sampled in summer (48,227.963 ± 584.70 mg/kg). The lowest Ca values were observed in *D. dentex* (11,388.46 ± 651.8 mg/kg) and *S. aurata* (13,232.30 ± 148.90 mg/kg) sampled in spring and in *D. sargus* (12,658.49 ± 1294.90 mg/kg) sampled in autumn. The highest K value was found in *S. aurata* sampled in summer (27,594.86 ± 732.80 mg/kg). The K values of the species *O. melanura*, *D. sargus, P. major, D. dentex* varied seasonally between 17,230.83–24,940.00 mg/kg. The lowest Mg value was found in *D. sargus* sampled in summer (1436.02 ± 87.50 mg/kg) and the highest value was found in *D. dentex* sampled in summer (2326.73 ± 225.38 mg/kg). While no statistical difference was observed between fall, summer and winter seasons (*p* > 0.05), a statistical difference was observed between spring and other seasons (*p* < 0.05). The highest Na value was found in *D. dentex* sampled in summer (7847.41 ± 760.20 mg/kg), while the lowest value was observed in *D. sargus* sampled in spring (1962.30 ± 22.11 mg/kg). The highest P values were observed in *D. sargus* (15,516.57 ± 7010.7 mg/kg) and (14,111.84 ± 1495.60 mg/kg) sampled in autumn, *S. aurata* (13,112.11 ± 454.20 mg/kg) sampled in summer and *P. major* (13,890.60 ± 1256.40 mg/kg) sampled in spring. Seasonal differences in elemental composition may be influenced by water temperature, fish diet, and metabolic differences.

#### 3.1.2. Trace Elements

The levels of trace elements, including Cu, Fe, Mn, Zn, V, Se, Ni, and Cr, were measured in fish species (Table 3). The lowest Cu value was observed in *P. major* during autumn (36.44 ± 1.58 mg/kg), while the highest Cu value was recorded in *D. dentex* during winter (59.13 ± 28.26 mg/kg). No significant seasonal differences were found in Cu values (*p* > 0.05). Copper is relatively safe for organisms and is an essential element in hemoglobin and certain enzymes. Therefore, EU and Turkish Food Codex regulations do not specify permissible limits for essential heavy metals like Cu [31]. However, excessive intake can lead to physical and psychiatric disorders such as kidney and liver diseases [32,33,34].

The lowest Fe value was observed in *S. aurata* during autumn (109.10 ± 13.2 mg/kg). The Fe values in *P. major, D. dentex, D. sargus*, and *O. melanura* ranged between 107.61 and 265.38 mg/kg across seasons. The lowest Mn values were observed in *O. melanura* during winter (6.76 ± 0.77 mg/kg) and *D. dentex* during spring (6.19 ± 0.98 mg/kg), while the highest Mn values were recorded in *D. dentex* during winter (19.87 ± 1.21 mg/kg) and autumn (17.23 ± 1.34 mg/kg). According to the Turkish Food Codex, the recommended allowable limit for Mn in fish species is 20 mg/kg [35]. In this study, all species were below the allowable limit for Mn across all seasons, except for *D. dentex* during winter, where Mn values approached the limit. Although low levels of manganese are essential for human health, excessive intake is toxic and can cause neurological adverse effects [36]. High concentrations of Mn can bypass liver metabolism and enter brain tissues, causing tremors, difficulty walking, and facial spasms [36]. While no carcinogenic effects of Mn have been reported, the US EPA’s accepted RfD is 140 µg/kg/day body weight [30]. Mn concentrations in this study were higher than those reported by Capodiferro et al. [37] in the western Mediterranean, suggesting regional differences. High Mn levels in fish tissues can be attributed to local sediment composition and river inflows.

Zn levels ranged between 98.67 ± 5.13 and 554.42 ± 95.74 mg/kg. The lowest Zn value was observed in *D. dentex* during spring, while the highest Zn levels were recorded in *D. sargus* during summer and in *P. major* during spring and winter. Zinc is an essential microelement crucial for humans and animals, acting as a cofactor in approximately 300 enzymes in mammalian organisms. High zinc levels are required to maintain specific biological functions. Zn deficiency can result in loss of appetite, impaired intelligence, skin changes, and immune system alterations [38]. Excess zinc intake can lead to acute adverse effects and organizational disorders [39]. Our findings on Cu and Zn align with Döndü et al. [40] and Lounas et al. [41], who reported similar levels in *S. aurata* from polluted coastal areas. Elevated Cu and Zn levels are linked to anthropogenic activities such as industrial runoff, which is also likely affecting Bozcaada’s waters.

Seasonal Ni values ranged between 6.33 ± 0.58 and 18.95 ± 1.31 mg/kg. The lowest Ni values were recorded in *D. dentex* during winter (6.33 ± 0.58 mg/kg) and *D. sargus* during summer (7.94 ± 1.88 mg/kg). The highest Ni values were observed in *D. dentex* during winter (13.89 ± 0.84 mg/kg) and *O. melanura* during summer (13.25 ± 1.38 mg/kg). V levels ranged seasonally from 1.00 ± 0.36 to 480 ± 0.37 mg/kg. The lowest V values were found in *D. dentex* during summer (1.10 ± 0.18 mg/kg) and spring (1.00 ± 0.36 mg/kg). Vanadium (V) is an essential element for some enzyme components and cell growth. While V is toxic at high concentrations, it has been shown to reduce the progression of diabetes and cancer cells. Vanadium has not been classified for allowable limits by USEPA and WHO, with a predicted daily upper limit of 0.2 mg/day [28].

In this study, seasonal Se values ranged from 0.82 ± 0.07 to 7.33 ± 0.04 mg/kg. Selenium is a crucial trace element for human health and nutrition. It interacts with mercury toxicity through high binding capacities between selenol groups of selenoproteins and exhibits antioxidant effects against reactive oxygen species induced by mercury. Selenium is vital for defending cells and organisms against oxidative damage. Se has gained importance in dietary supplements due to its effects on treating inflammation, rejuvenating an aging immune system, and protecting against cancer development [42,43]. For these reasons, seafood is considered an excellent selenium source.

Seasonal Cr levels ranged between 3.60 ± 0.24 and 15.34 ± 3.57 mg/kg. The highest Cr value was observed in *P. major* during summer (11.96 ± 0.56 mg/kg) and *D. dentex* during winter (11.34 ± 1.46 mg/kg). In *P. major*, Cr values peaked during summer and decreased in autumn (10.73 ± 0.24 mg/kg), winter (9.76 ± 0.92 mg/kg), and spring (7.21 ± 1.37 mg/kg). Statistically significant differences were observed between spring and other seasons (*p* < 0.05), while no differences were noted between autumn, winter, and summer (*p* > 0.05). Se levels were consistent with Solé et al. [44], who emphasized its protective role against mercury toxicity. Elevated Ni and Cr levels observed in summer align with findings by Lounas et al. [41] and Döndü et al. [40], reflecting the impact of industrial activities and seasonal bioaccumulation.

#### 3.1.3. Toxic Metals

The levels of toxic metals Cd and Hg were investigated in five different sparid species caught in Bozcaada (Table 2). Cadmium is a significant contaminant that can be transported through water and air and is a highly toxic element found in various sources. As a non-essential element, Cd has toxic effects on kidney functions and bones and is associated with prostate cancer [45,46]. Cd is generally found in low concentrations in aquatic environments. Our findings align with those of Döndü et al. [40] and Lounas et al. [41], who reported elevated Cd and Hg concentrations in S. aurata from polluted coastal areas.

Cd levels in fish species ranged from 0.04 ± 0.05 to 0.55 ± 0.07 mg/kg. The lowest Cd levels were observed in *D. sargus* (0.08 ± 0.00 mg/kg) and *O. melanura* (0.08 ± 0.00 mg/kg) during spring, and in *D. dentex* (0.09 ± 0.01 mg/kg) during winter. The highest Cd levels were found in *O. melanura* during autumn (0.32 ± 0.07 mg/kg). In *D. sargus*, Cd levels increased from 0.08 ± 0.00 mg/kg in spring to 0.10 ± 0.01 mg/kg in winter, 0.20 ± 0.03 mg/kg in autumn, and 0.19 ± 0.04 mg/kg in summer. No statistical differences were observed between spring and winter or autumn and summer (*p* > 0.05). In *O. melanura*, Cd levels increased from 0.08 ± 0.00 mg/kg in spring to 0.14 ± 0.02 mg/kg in winter, 0.24 ± 0.06 mg/kg in summer, and 0.32 ± 0.07 mg/kg in autumn. Statistical differences were found between autumn and summer, summer and winter, and winter and spring (*p* < 0.05). In *D. dentex*, Cd levels increased from 0.09 ± 0.01 mg/kg in winter to 0.14 ± 0.01 mg/kg in spring, 0.19 ± 0.01 mg/kg in summer, and 0.28 ± 0.01 mg/kg in autumn. No statistical differences were observed between seasons (*p* > 0.05). According to the Turkish Food Codex (2011), EU regulations (2006), and WHO guidelines (2011), the maximum allowable limit for cadmium in fish is 0.05 mg/kg. In this study, it was observed that the Cd element content of the species in all seasons was detected above the limits set by the authorities. Cd levels observed in this study were similar to those reported by Capodiferro et al. [37] in the Tyrrhenian Sea, indicating that Cd contamination is a widespread issue in Mediterranean fisheries. Seasonal peaks in Hg levels, particularly during summer, reflect findings by Solé et al. [44], who noted that warmer temperatures and water evaporation can increase metal bioavailability in aquatic environments.

Mercury, another toxic element investigated, is a highly toxic metal considered a global pollutant with significant environmental risks. Hg is easily absorbed by the human body and can damage the central nervous system. Seafood, especially filter-feeding species like mussels and oysters, is thought to be the primary source of Hg in the human food chain. Therefore, seafood is an important indicator of Hg pollution in marine ecosystems [47]. Seafood is also a significant pathway for exposure to methylmercury [48]. The highest Hg level was found in *D. sargus* during summer (1.50 ± 0.27 mg/kg), while the lowest levels were observed in *D. dentex* during summer (0.08 ± 0.02 mg/kg) and autumn (0.14 ± 0.00 mg/kg), and in *S. aurata* during autumn (0.19 ± 0.01 mg/kg). In *D. sargus*, Hg levels decreased from 1.50 ± 0.27 mg/kg in summer to 0.89 ± 0.04 mg/kg in winter, 0.32 ± 0.03 mg/kg in spring, and 0.31 ± 0.01 mg/kg in autumn. No statistical differences were observed between spring and autumn (*p* > 0.05), but differences were noted with other seasons (*p* < 0.05). In *D. dentex*, Hg levels increased from 0.08 ± 0.02 mg/kg in summer to 0.14 ± 0.00 mg/kg in autumn, 0.26 ± 0.10 mg/kg in winter, and 0.73 ± 0.04 mg/kg in spring. No statistical differences were observed between autumn, winter, and spring (*p* > 0.05). The Hg was not detected in *P. major* samples during spring, winter, and autumn, likely due to natural variations, environmental factors, and the species’ trophic level. Seasonal changes in water quality, fish age, feeding habits, and analytical detection limits (ICP-MS) may all contribute to this result. These factors combined suggest that mercury levels were below detection thresholds, and further studies should ensure continued monitoring for fish safety assessments.

Among metals, Hg is the most toxic, with the Turkish Food Codex and European Commission Regulation recommending an upper limit of 0.5 mg/kg [31]. Methylmercury toxicity involves effects such as inhibition of protein synthesis, microtubule disruption, neurotransmitter dysfunction, and intracellular calcium increase. According to the Turkish Food Codex, Hg levels in *O. melanura* during winter, *D. sargus* during spring and autumn, and *D. dentex* during winter, autumn, and summer were below the maximum permissible limit and deemed safe for consumption.

The studied species exhibited seasonal variations in their elemental composition. Multiple studies showed that metal accumulation in aquatic organisms is influenced by factors such as metal concentration, water temperature, salinity, depth, species, gender, size, weight, and age [49,50]. Increasing metal concentrations in aquatic environments affect various tissues and organs of aquatic organisms, including fish, and accumulate in plant-based and animal-based marine organisms consumed by humans. Metals entering the body bind to carrier proteins and are transported through the bloodstream to tissues and organs, where they reach high concentrations by binding to metal-binding proteins. Elevated metal concentrations in aquatic environments pose threats to aquatic ecosystems and human health. The consumption of fish muscle tissue by humans facilitates the transfer of metals due to fish being at the top of the aquatic food chain [51,52]. Studies have shown that bioaccumulation increases with the age and weight of aquatic organisms [5,53,54]. The toxic metal accumulation levels detected in fish muscle tissue based on age and weight may have adverse effects on human health. Larger fish, often preferred for consumption, are thought to lead to greater heavy metal accumulation in humans due to their higher temporal and dietary metal exposure. Fish species with different physiological structures can accumulate metals in varying amounts in different tissues based on exposure duration and the type of metal [53]. Considering that the studied species weighed between 500–600 g, it is believed that this might influence the high elemental content and bioaccumulation in muscle tissue. Studies have also highlighted that factors such as season, temperature, and evaporation influence the metal content of fish muscle tissue. Kır et al. [55] reported that metal concentrations in water increased during summer due to evaporation, leading to higher metal accumulation in sediment. In spring, lower levels were attributed to water circulation. However, Özmen et al. [56] found the highest metal content in muscle tissue during spring. Seasonal differences in elemental content were observed in this study. The dietary habits of fish also play a crucial role in influencing the elemental composition of muscle tissue. Kalay et al. [57] compared cadmium levels in muscle and liver tissues of *S. aurata* and *Mullus barbatus* caught in Mersin Bay, noting cadmium levels exceeding permissible limits, indicating heavy metal contamination in the region. High cadmium concentrations were partially attributed to dietary sources, such as small invertebrates, fish larvae, mollusks, crustaceans, detritus, and worms. These aquatic organisms are known for their high levels of heavy metal accumulation.

### 3.2. Consumer Health Risk Assessment

As part of the consumer health risk assessment, values for EWI (Estimated Weekly Intake), THQ (Target Hazard Quotient), and CR (Lifetime Cancer Risk) were calculated. These calculations were based on the assumption that the five species obtained from the Bozcaada region were consumed by adult and child consumers for 1, 3, and 5 days per week in their meals.

#### 3.2.1. Estimated Weekly Intake (EWI)

The EWI values of all metals measured in five fish species from Bozcaada were lower than the temporary PTWI limits set by the authorities (Table 4). Estimated weekly intake values were compared with PTWI (Provisional Tolerable Weekly Intake) values. The PTWI values for Cu and Fe [58], Zn (JECFA 2010) [59], Hg (WHO/FAO 2011) [60], Ni (JECFA 2011) [61], (EFSA 2009) [62] were reported as 125, 5600, 300–1000, 4, 5000, and 15 (µg/kg), respectively. The PTWI value for Cd, which has a Provisional Monthly Tolerable Intake (PTMI) of 25 (µg/kg), was reported as 6.25 (µg/kg) (JECFA 2013) [63]. Tolerable daily intake (TDI) values for Mn (USEPA 2019) [30] and Cr (EFSA 2014) [63] were reported as 140 and 300 (µg/kg/day), respectively. Based on these values and individual body weights, the weekly intake limits for Cu, Fe, Mn, Zn, Hg, Ni, Cr, and Cd were determined as 8750, 392,000, 68,600, 21,000–70,000, 280, 350,000, 147,000, and 437.5 (µg/kg) for adults; and 4000, 179,200, 31,360, 9600–32,000, 128, 160,000, 67,200, and 200 (µg/kg) for children, respectively.

When comparing EWI values with PTWI values for Cu, Fe, Mn, Zn, Hg, Ni, Cr, and Cd elements among adults and children consuming the examined species 1, 3, and 5 days per week, it was found that EWI values for all elements were below PTWI limits. Storelli et al. [64] evaluated EWI values for Hg, Pb, and Cd in seafood (fish, cephalopods, and shrimp) from the Adriatic Sea. They found that while EWI values for cephalopods and shrimp were below the limits for Hg, fish EWI values exceeded the limits, posing a potential risk. Similarly, Storelli et al. [64] found that the EWI value for Cd in *Mustela mustela* species from the Mediterranean Sea was below the limits set by authorities. Mol et al. [65] evaluated the health risks of heavy metals for Turkish and Greek consumers of spiny dogfish and rays from the Marmara Sea, reporting that Cu, Zn, Cd, and Hg intakes were below the tolerable weekly intake limits for both countries. Korkmaz et al. [66] found that EWI values for Cr, Mn, Fe, Ni, Cu, Zn, and Pb in 16 different economic fish species from Taşucu and Mersin regions were below the tolerable limits set by US EPA. Köşker [15] showed that EWI values for Cr, Cd, and Pb in six economic fish species from Mersin Bay, consumed by adults and children for 1, 3, and 7 days per week, were below the PTWI limits set by FAO/WHO and European Food Safety Authority. Turanlı and Gedik [50] found that EWI values for *Mytilus galloprovincialis* species from the Black Sea, Marmara, and Aegean Sea were below limits for elements like As, Cd, Cr, Cu, Ni, Pb, V, and Zn. In this study, weekly EWI values showed no potential risk when adults consumed all species for 1, 3, and 5 days per week, and children consumed them for 1 day per week. However, children consuming all species for 3 days per week during seasonal periods showed potential risk limits for Cu and Zn. Consumption for 5 days per week indicated a potential risk. Our results align with those of Döndü et al. [40] and Lounas et al. [41], who reported that EWI values for Cd and Hg in *S. aurata* were within safe limits for most consumption scenarios. However, they also noted potential risks when consumption frequency increases, particularly for children.

#### 3.2.2. Target Hazard Quotient (THQ)

THQ, or HQ (Hazard Quotient), is a quantitative assessment method used to evaluate non-cancer health risks, especially when exposure to multiple chemicals occurs simultaneously. In this study, THQ was used to determine the potential hazard of metal contaminants in seafood from the Bozcaada region for adults and children (Table 5). A THQ value > 1 indicates that the metal level is ≥RfD, signifying potential health risks. In this study, seasonal non-carcinogenic risk levels for all species showed THQ < 1, except for seasonal THQ > 1 values for Ni and Hg as consumption frequency increased. Similar trends were observed in other studies.

For Fe, Mn, Zn, and Cu, all seasonal THQ values were <1 for both children and adults consuming *D. dentex*, indicating no risk. However, in summer, adult THQ values for V exceeded the threshold (THQ > 1) when consumed 5 days per week, and for children consuming *D. dentex* for 3 and 5 days. THQSe values remained <1 during winter and autumn for all species. Seasonal THQCd and THQCr values were <1, posing no risk. Similarly, THQAs values were <1 for all species across all seasons. THQHg values for adults consuming *D. dentex* for 1-, 3-, and 5-days during winter, autumn, and summer were <1. THQNi values were <1 for adults consuming *D. dentex* during spring and summer for 1, 3, and 5 days, while children showed THQNi < 1 during the same periods. (Table 4). Our findings are consistent with Döndü et al. [40], who reported THQ values for Cd and Hg below critical thresholds for *S. aurata* from polluted coastal areas. Similarly, Lounas et al. [41] noted that THQ values for Hg in wild-caught fish were close to risk thresholds in some Mediterranean regions. This aligns with our findings, where THQ values for Hg approached 1 during summer, particularly for frequent consumers. Capodiferro et al. [37] emphasized that seasonal variations in metal levels directly affect THQ values, with summer showing the highest risk due to increased bioaccumulation. Our results follow a similar trend, highlighting that regular consumption of fish with elevated metal levels during certain seasons may pose health risks.

#### 3.2.3. Lifetime Cancer Risk (CR)

To evaluate cancer risks associated with consuming different aquatic species around Bozcaada, CR values were calculated for Cr and Cd (Table 6). The healthy lifetime cancer risk probability is set at 10^−5^. CR values < 10^−5^ are considered safe [30]. According to USEPA, CR values below 10^−6^ are “negligible”, values above 10^−4^ are “unacceptable”, and values between 10^−6^ and 10^−4^ are “acceptable.” CR values were calculated for high consumption rates.

For Cd, CR values exceeded the threshold (CR > 10^−5^) when adults and children consumed *P. major* and *D. dentex* for 5 days in all seasons, and *D. sargus* during autumn, winter, and summer. For single-day weekly consumption, CR values for all species remained below the threshold (CR < 10^−5^), indicating no carcinogenic risk. Similarly, CR values for *D. dentex* remained below the threshold for single-day weekly consumption across all seasons.

Our findings are in line with Döndü et al. [40], who reported CR values below the acceptable limit for *S. aurata* from the Güllük Lagoon. However, they emphasized that long-term consumption of fish from polluted areas could result in elevated cancer risks, particularly for children. Similarly, Lounas et al. [41] found that the CR values for Cd in wild fish from the southern Mediterranean were close to risk thresholds for frequent consumers. Our results show a similar pattern, especially for children consuming fish with higher metal levels during certain seasons. Capodiferro et al. [40] highlighted that CR values vary significantly with seasonal fluctuations in metal concentrations. Our study reflects this, with CR values for Cd exceeding safe limits in some cases during summer and autumn. These findings underline the importance of regulating fish consumption frequency and continuously monitoring metal levels to minimize cancer risks. Uçar [67] found that CR values for Cr in *Boops boops* species from Mersin Bay were below the threshold, posing no carcinogenic risk for weekly consumption of 1, 3, or 7 days. However, for Cd, as consumption increased to 7 days, CR values exceeded the threshold (CR >10^−5^). Köşker [15] showed that CR values for Cr in *M. barbatus, S. lesepsianus, C. lucerna, T. mediterraneus, P. erythrinus, M. japonicus*, and *P. longirostris* remained below the threshold for adult consumption. For children, CR values for 1 and 3-day weekly consumption were below the threshold, but 7-day weekly consumption for certain species exceeded the threshold (CR > 10^−5^), posing a carcinogenic risk.

## 4. Conclusions

The findings demonstrate that while the concentrations of essential minerals, such as calcium, magnesium, and zinc, were within safe limits for human consumption, seasonal variations in the levels of toxic metals, including Hg and Cd, could pose potential health risks, particularly with frequent consumption. The health risk assessment conducted using the EWI, THQ, and CR values indicated that, in general, the fish species studied posed no significant health risks for adults consuming them up to five times per week. However, for children, there were potential risks from certain metals, especially with increased consumption frequency, particularly for Cu and Zn.

The results emphasize the importance of considering both the nutritional benefits and the risks associated with toxic metal exposure when evaluating fish consumption. These findings provide valuable insights into the safety of seafood consumption in the region and highlight the significance of continuous monitoring of seasonal changes. Future studies should continue to monitor these seasonal changes and evaluate their long-term health impacts to ensure that consumption remains within safe limits for all age groups.

## Figures and Tables

**Table 1:** Morphometric data (weight and length) of investigated sparid species.

Scientific Name	Family	Common Name	*n*	Weight (g)	Lenght (cm)
*Pagrus major* (Temminck & Schlegel, 1843)	*Sparidae*	Red seabream	40	246.07 ± 6.58	31.13 ± 0.45
*Sparus aurata* (Linnaeus, 1758)	*Sparidae*	Gilthead seabream	40	230.04 ± 24.26	36.91 ± 1.54
*Oblada melanura* (Linnaeus, 1758)	*Sparidae*	Saddled seabream	40	696.99 ±33.73	26.97 ± 1.37
*Diplodus sargus* (Linnaeus, 1758)	*Sparidae*	White seabream	40	390.91 ± 23.75	33.79 ± 1.58
*Dentex dentex* (Linnaeus, 1758)	*Sparidae*	Common dentex	40	688.53 ± 20.97	32.23 ± 1.05

**Table 2:** Seasonal changes of macro elemet composition in Sparidae species (mg/kg).

Species	Seasons	Ca	K	Mg	Na	P
*O. melanura*	Spring	22,768.60 ± 483.1 ^b^	20,803.98 ± 988.0 ^b^	1889.51 ± 91.8 ^bc^	4191.03 ± 746.6 ^b^	11,278.68 ± 350.32 ^ab^
	Winter	19,200.27 ± 1064.13 ^b^	22,598.45 ± 118.80 ^ab^	1769.63 ± 90.67 ^c^	2462.96 ± 27.40 ^c^	12,597.17 ± 755.67 ^ab^
	Autumn	33,364.77 ± 4643.7 ^a^	23,225.95 ± 1037.9 ^a^	1974.60 ± 7.93 ^b^	4275.75 ± 33.55 ^b^	14,111.84 ± 1495.6 ^a^
	Summer	26,026.77 ± 1957.9 ^b^	17,230.83 ± 566.8 ^c^	2180.11 ± 20.0 ^a^	7129.75 ± 500.0 ^a^	10,501.15 ± 1281.7 ^b^
*S. aurata*	Spring	13,232.31 ± 148.9 ^b^	21,443.26 ± 323.8 ^b^	1617.60 ± 39.37 ^b^	2872.38 ± 43.47 ^b^	10,909.07 ± 278.0 ^b^
	Winter	16,821.90 ± 169.9 ^ab^	24,620.89 ± 412.4 ^ab^	1758.75 ± 26.5 ^b^	2894.92 ± 42.3 ^b^	11,909.80 ± 297.5 ^ab^
	Autumn	17,552.32 ± 1952.4 ^ab^	18,142.41 ± 2145.2 ^c^	1569.58 ± 127.6 ^b^	2652.44 ± 62.5 ^b^	8996.80 ± 829.8 ^c^
	Summer	21,167.55 ± 2386.8 ^a^	27,594.86 ± 732.8 ^a^	2003.91 ± 58.2 ^a^	4440.33 ± 408.5 ^a^	13,112.11 ± 454.2 ^a^
*D. sargus*	Spring	23,491.83 ± 1238.9 ^a^	20,451.33 ± 455.5 ^a^	1497.84 ± 175.6 ^b^	1962.30 ± 22.11 ^c^	10,653.75 ± 635.7 ^a^
	Winter	15,933.30 ± 103.7 ^c^	20,813.34 ± 55.11 ^a^	1700.34 ± 3.11 ^b^	3876.59 ± 43.6 ^b^	9679.20 ± 329.2 ^a^
	Autumn	12,658.49 ± 1294.9 ^d^	20,286.55 ± 139.4 ^a^	2036.19 ± 68.3 ^a^	5564.16 ± 168.0 ^a^	15,516.57 ± 7010.7 ^a^
	Summer	19,800.93 ± 309.3 ^b^	20,688.24 ± 1562.8 ^a^	1436.02 ± 87.5 ^b^	3839.95 ± 57.6 ^b^	10,569.60 ± 593.1 ^a^
*P. major*	Spring	52,357.53 ± 2927.6 ^a^	20,598.66 ± 519.1 ^a^	1763.91 ± 36.0 ^a^	4419.10 ± 208.3 ^a^	13,890.60 ± 1256.4 ^a^
	Winter	55,470.76 ± 1277.4 ^a^	23,960.86 ± 1306.3 ^a^	1861.73 ± 24.91 ^a^	4309.28 ± 141.5 ^a^	13,932.89 ± 909.0 ^a^
	Autumn	15,543.13 ± 956.8 ^b^	22,256.24 ± 927.2 ^a^	1554.95 ± 78.8 ^a^	3609.39 ± 30.13 ^ab^	11,391.74 ± 905.8 ^a^
	Summer	17,843.79 ± 20.62 ^b^	24,365.49 ± 5478.3 ^a^	1625.77 ± 488.9 ^a^	2835.00 ± 745.1 ^b^	13,020.81 ± 492.5 ^a^
*D. dentex*	Spring	11,388.46 ± 651.8 ^d^	23,787.12 ± 2269.3 ^a^	1655.61 ± 72.6 ^b^	2871.80 ± 133.2 ^c^	10,928.59 ± 1004.1 ^a^
	Winter	17,172.32 ± 1519.2 ^c^	22,250.30 ± 1672.6 ^a^	1611.33 ± 153.8 ^b^	2163.42 ± 101.3 ^c^	10,759.13 ± 481.1 ^a^
	Autumn	22,557.39 ± 92.90 ^b^	22,970.18 ± 194.56 ^a^	1506.72 ± 8.34 ^b^	4521.77 ± 98.64 ^b^	10,612.18 ± 27.8 ^a^
	Summer	48,227.93 ± 584.7 ^a^	24,940.93 ± 2805.4 ^a^	2326.73 ± 225.38 ^a^	7847.41 ± 760.2 ^a^	12,717.43 ± 1279.4 ^a^

Different letters (^a, b, c^) in the same columns for each season indicate significant differences (*p* < 0.05).

**Table 3:** Seasonal changes of trace element and toxic metal composition in Sparidae species (mg/kg).

Species	Seasons	Cu	Fe	Mn	Zn	V	Se	Ni	Cr	Cd	Hg
*O. melanura*	Spring	43.17 ± 3.02 ^a^	116.82 ± 20.39 ^a^	12.60 ± 0.12 ^a^	164.97 ± 40.30 ^a^	1.92 ± 0.13 ^a^	2.84 ± 0.03 ^a^	10.81 ± 0.94 ^b^	4.54 ± 0.65 ^a^	0.08 ± 0.00 ^c^	0.78 ± 0.08 ^a^
	Winter	45.97 ± 3.51 ^a^	122.49 ± 23.26 ^a^	6.76 ± 0.77 ^c^	119.09 ± 36.0 ^a^	1.96 ± 0.36 ^a^	2.22 ± 1.85 ^a^	8.33 ± 0.21 ^c^	4.94 ± 0.22 ^a^	0.14 ± 0.02 ^bc^	0.41 ± 0.07 ^c^
	Autumn	53.41 ± 8.03 ^a^	153.88 ± 67.61 ^a^	12.44 ± 0.32 ^a^	215.23 ± 75.61 ^a^	2.08 ± 0.44 ^a^	6.48 ± 3.11 ^a^	11.41 ± 0.24 ^ab^	4.34 ± 0.026 ^a^	0.32 ± 0.07 ^a^	0.66 ± 0.02 ^ab^
	Summer	41.56 ± 10.4 ^a^	136.24 ± 33.09 ^a^	9.94 ± 0.05 ^b^	256.26 ± 220.3 ^a^	2.10 ± 0.03 ^a^	3.87 ± 3.45 ^a^	13.25 ± 1.38 ^a^	5.79 ± 2.21 ^a^	0.24 ± 0.06 ^ab^	0.51 ± 0.03 ^bc^
*S. aurata*	Spring	43.75 ± 2.34 ^a^	212.24 ± 10.50 ^a^	9.43 ± 1.72 ^b^	136.49 ± 17.4 ^a^	1.88 ± 0.05 ^a^	2.52 ± 1.30 ^b^	9.46 ± 0.82 ^a^	7.19 ± 0.00 ^a^	0.18 ± 0.04 ^ab^	0.62 ± 0.00 ^a^
	Winter	42.69 ± 8.96 ^a^	136.35 ± 1.36 ^b^	7.36 ± 0.20 ^b^	175.64 ± 65.8 ^a^	2.16 ± 0.34 ^a^	2.17 ± 0.33 ^b^	11.47 ± 3.95 ^a^	5.74 ± 0.86 ^a^	0.11 ± 0.00^c^	0.70 ± 0.03 ^a^
	Autumn	44.48 ± 0.98 ^a^	109.10 ± 13.2 ^b^	8.17 ± 0.06 ^b^	140.36 ± 30.43 ^a^	1.73 ± 0.00 ^a^	1.38 ± 0.13 ^b^	9.39 ± 0.04 ^a^	6.84 ± 0.62 ^a^	0.21 ± 0.01 ^a^	0.19 ± 0.01 ^b^
	Summer	53.08 ± 4.73 ^a^	222.73 ± 17.86 ^a^	14.18 ± 0.05 ^a^	281.68 ± 153.68 ^a^	1.95 ± 0.08 ^a^	4.52 ± 0.47 ^a^	10.48 ± 1.99 ^a^	6.57 ± 0.03 ^a^	0.17 ± 0.03 ^ab^	0.67 ± 0.06 ^a^
*D. sargus*	Spring	46.03 ± 8.72 ^a^	170.46 ± 7.16 ^b^	12.04 ± 6.98 ^a^	136.87 ± 51.2 ^b^	1.52 ± 0.65 ^a^	2.37 ± 0.70 ^b^	8.45 ± 0.35 ^a^	10.86 ± 0.012 ^a^	0.08 ± 0.00 ^b^	0.32 ± 0.03 ^c^
	Winter	46.41 ± 5.77 ^a^	249.06 ± 8.98 ^a^	15.85 ± 0.26 ^a^	378.40 ± 164.1 ^ab^	2.27 ± 0.90 ^a^	5.50 ± 1.43 ^a^	10.11 ± 0.78 ^a^	9.77 ± 0.42 ^bc^	0.10 ± 0.01 ^b^	0.89 ± 0.04 ^b^
	Autumn	42.85 ± 4.62 ^a^	262.76 ± 32.21 ^a^	11.88 ± 0.25 ^a^	136.50 ± 40.2 ^b^	2.43 ± 0.11 ^a^	1.16 ± 0.05 ^b^	9.55 ± 1.24 ^a^	9.05 ± 0.52 ^c^	0.20 ± 0.03 ^a^	0.31 ± 0.01 ^c^
	Summer	43.58 ± 2.66 ^a^	198.26 ± 33.44 ^ab^	12.68 ± 0.03 ^a^	554.42 ± 95.74 ^a^	1.82 ± 0.29 ^a^	6.77 ± 0.17 ^a^	7.94 ± 1.88 ^a^	10.47 ± 0.02 ^ab^	0.19 ± 0.04 ^a^	1.50 ± 0.27 ^a^
*P. major*	Spring	39.70 ± 1.67 ^a^	145.14 ± 27.60 ^b^	12.81 ± 2.09 ^a^	421.23 ± 6.35 ^a^	1.87 ± 0.10 ^a^	6.53 ± 0.17 ^a^	9.50 ± 1.09 ^ab^	7.21 ± 1.37 ^b^	0.12 ± 0.05 ^b^	0.15 ± 0.01
	Winter	47.82 ± 5.51 ^a^	265.38 ± 4.11 ^a^	14.80 ± 0.40 ^a^	451.14 ± 19.95 ^a^	1.72 ± 0.05 ^a^	6.62 ± 0.28 ^a^	12.17 ± 1.76 ^a^	9.76 ± 0.92 ^a^	0.24 ± 0.02 ^a^	ND
	Autumn	36.44 ± 1.58 ^a^	183.13 ± 17.2 ^b^	11.27 ± 0.31 ^a^	184.41 ± 15.68 ^b^	1.81 ± 0.35 ^a^	3.74 ± 0.55 ^a^	8.90 ± 0.77 ^b^	10.73 ± 0.24 ^a^	0.14 ± 0.01 ^b^	ND
	Summer	40.85 ± 7.77 ^a^	202.00 ± 26.3 ^b^	11.19 ± 1.80 ^a^	209.98 ± 11.4 ^b^	1.84 ± 0.01 ^a^	3.52 ± 3.40 ^a^	9.24 ± 0.58 ^ab^	11.96 ± 0.56 ^a^	0.12 ± 0.00 ^b^	ND
*D. dentex*	Spring	41.61 ± 4.20 ^a^	127.18 ± 17.29 ^b^	6.19 ± 0.98 ^c^	105.91 ± 9.22 ^c^	1.00 ± 0.36 ^b^	1.54 ± 0.01 ^a^	6.33 ± 0.58 ^c^	7.73 ± 1.26 ^b^	0.14 ± 0.01 ^c^	0.73 ± 0.04 ^a^
	Winter	59.13 ± 28.26 ^a^	256.46 ± 12.94 ^a^	19.87 ± 1.21 ^a^	235.45 ± 4.57 ^a^	1.40 ± 0.24 ^ab^	3.5 ± 1.09 ^a^	13.89 ± 0.84 ^a^	11.34 ± 1.46 ^a^	0.09 ± 0.01 ^d^	0.26 ± 0.10 ^b^
	Autumn	47.91 ± 8.10 ^a^	282.00 ± 23.9 ^a^	17.23 ± 1.34 ^a^	209.82 ± 3.06 ^b^	1.809 ± 0.17 ^a^	3.45 ± 1.93 ^a^	13.12 ± 1.00 ^ab^	9.40 ± 0.22 ^ab^	0.28 ± 0.01 ^a^	0.14 ± 0.00 ^ab^
	Summer	42.91 ± 1.85 ^a^	281.91 ± 10.74 ^a^	10.29 ± 0.87 ^b^	200.55 ± 4.31 ^b^	1.10 ± 0.18 ^b^	3.34 ± 0.17 ^a^	10.61 ± 1.56 ^b^	10.70 ± 1.40 ^ab^	0.19 ± 0.002 ^b^	0.08 ± 0.02 ^c^

Different letters (^a, b, c^) in the same columns for each season indicate significant differences (*p* < 0.05).

**Table 4:** Estimated Weekly Intake (EWI; μg/kg BW) values for each metal analyzed according to different consumption frequencies.

		*O. melanura*		*S. aurata*		*D. dentex*		*P. major*		*D. sargus*	
	Day/ Week	A	C	A	C	A	C	A	C	A	C
Cu	5	84.49	184.82	84.43	137.19	94.18	135.93	75.63	121.95	96.30	124.15
	3	36.21	79.21	36.19	58.90	40.47	58.36	32.41	52.36	41.37	53.31
	1	12.07	26.40	12.06	19.63	13.49	19.45	10.81	17.45	13.79	17.77
Fe	5	242.96	531.47	312.24	477.97	367.66	574.21	365.13	505.96	384.53	519.06
	3	104.13	227.78	133.82	204.95	157.68	246.20	156.48	216.94	164.90	223.66
	1	34.71	75.92	44.61	68.32	52.56	82.06	52.16	72.31	54.97	74.55
Mn	5	19.16	41.90	18.42	30.70	23.72	35.40	25.27	35.06	25.46	33.62
	3	8.21	17.96	7.89	13.27	10.28	15.28	10.83	15.12	11.01	14.51
	1	2.74	5.99	2.63	4.42	3.42	5.09	3.61	5.04	3.67	4.84
Zn	5	346.72	758.46	336.92	549.38	374.13	536.90	741.94	845.12	516.05	703.86
	3	148.60	325.05	144.26	235.54	160.43	230.19	317.97	362.27	221.25	301.73
	1	49.53	108.35	48.13	78.52	53.48	76.74	105.99	120.76	73.75	100.58
V	5	3.69	8.08	3.54	6.41	4.07	5.24	3.32	5.70	4.60	5.85
	3	1.58	3.46	1.52	2.86	1.80	2.35	1.42	2.54	2.07	2.60
	1	0.53	1.15	0.51	0.95	0.62	0.78	0.47	0.85	0.69	0.87
Se	5	7.07	15.47	4.87	9.37	7.60	10.12	9.36	12.57	8.49	10.96
	3	3.03	6.63	2.09	4.12	3.38	4.46	4.02	5.49	3.74	4.80
	1	1.01	2.21	0.70	1.37	1.13	1.49	1.34	1.83	1.25	1.60
Hg	5	1.11	2.42	1.00	2.29	1.56	1.83	0.07	1.32	1.70	2.17
	3	0.48	1.04	0.43	1.09	0.78	0.89	0.03	0.67	0.83	1.03
	1	0.16	0.35	0.14	0.36	0.26	0.30	0.01	0.22	0.28	0.34
Ni	5	20.11	43.98	41.67	59.42	27.87	37.45	18.27	34.47	27.26	32.87
	3	8.62	18.85	17.86	25.57	12.05	16.16	7.83	14.87	11.78	14.19
	1	2.87	6.28	5.95	8.52	3.95	5.38	2.61	4.95	3.92	4.72
Cr	5	9.00	19.69	12.09	18.94	15.18	23.71	18.20	23.42	16.84	23.09
	3	3.86	8.44	5.18	8.22	6.61	10.27	7.80	10.13	7.32	10.00
	1	1.29	2.81	1.73	2.74	2.20	3.42	2.60	3.38	2.44	3.33
Cd	5	0.36	0.78	0.31	1.14	0.99	1.14	0.28	1.04	0.92	1.00
	3	0.15	0.34	0.14	0.60	0.53	0.60	0.12	0.54	0.49	0.53
	1	0.05	0.11	0.05	0.20	0.18	0.20	0.04	0.18	0.16	0.18

A, adult; C, children. Exposure time of 5, 3 and 1 days/week.

**Table 5:** Target Hazard Quotient (THQ) values for each metal analyzed according to different consumption frequencies.

		*O. melanura*		*S. aurata*		*D. dentex*		*P. major*		*D. sargus*	
	Day/Week	A	C	A	C	A	C	A	C	A	C
Cu	5	0.21	0.47	0.22	0.97	0.86	0.97	0.19	0.88	0.82	0.89
	3	0.13	0.28	0.13	0.58	0.52	0.58	0.11	0.53	0.49	0.53
	1	0.04	0.09	0.04	0.19	0.17	0.19	0.04	0.18	0.16	0.18
Fe	5	0.04	0.08	0.05	0.69	0.68	0.71	0.05	0.65	0.63	0.65
	3	0.02	0.05	0.03	0.42	0.41	0.42	0.03	0.39	0.38	0.39
	1	0.01	0.02	0.01	0.14	0.14	0.14	0.01	0.13	0.13	0.13
Mn	5	0.02	0.03	0.01	0.65	0.86	0.67	0.02	0.62	0.61	0.62
	3	0.01	0.02	0.01	0.39	0.39	0.39	0.01	0.36	0.35	0.36
	1	0.00	0.01	0.00	0.13	0.13	0.13	0.00	0.12	0.12	0.12
Zn	5	0.12	0.26	0.12	0.81	0.75	0.81	0.25	0.86	0.75	0.81
	3	0.07	0.16	0.07	0.49	0.45	0.48	0.15	0.51	0.45	0.49
	1	0.02	0.05	0.03	0.16	0.15	0.16	0.05	0.17	0.15	0.16
V	5	0.08	0.17	0.07	0.74	0.91	1.17	0.07	0.73	0.71	0.74
	3	0.05	0.10	0.04	0.45	0.51	0.62	0.04	0.43	0.42	0.43
	1	0.02	0.03	0.01	0.15	0.17	0.21	0.01	0.14	0.14	0.14
Se	5	0.14	0.32	0.10	0.80	1.59	2.44	0.19	1.02	0.94	0.99
	3	0.09	0.19	0.06	0.48	0.83	1.19	0.12	0.58	0.53	0.56
	1	0.03	0.07	0.02	0.16	0.28	0.40	0.04	0.19	0.18	0.19
Hg	5	**1.13**	**2.46**	**1.02**	**2.31**	**1.58**	**1.85**	0.07	**1.34**	**1.72**	**2.20**
	3	0.68	**1.48**	0.61	**1.39**	0.94	**1.10**	0.04	0.80	**1.03**	**1.32**
	1	0.23	0.49	0.20	0.46	0.31	0.37	0.02	0.27	0.33	0.44
Ni	5	**1.03**	**2.24**	**2.12**	**3.62**	**2.01**	**2.50**	0.93	**2.30**	**1.93**	**2.22**
	3	0.62	**1.34**	**1.27**	**2.17**	**1.21**	**1.50**	0.56	**1.38**	**1.16**	**1.33**
	1	0.21	0.45	0.43	0.72	0.40	0.50	0.19	0.46	0.39	0.44
Cr	5	0.03	0.07	0.04	0.69	0.68	0.70	0.06	0.65	0.63	0.65
	3	0.02	0.04	0.03	0.41	0.41	0.42	0.04	0.39	0.38	0.39
	1	0.01	0.01	0.01	0.14	0.13	0.14	0.01	0.13	0.13	0.13
Cd	5	0.04	0.08	0.03	0.68	0.70	0.75	0.03	0.63	0.62	0.63
	3	0.03	0.05	0.02	0.41	0.41	0.44	0.02	0.38	0.37	0.37
	1	0.01	0.02	0.01	0.14	0.14	0.15	0.01	0.13	0.12	0.13

A, adult; C, children. Exposure time of 5, 3, and 1 days/week. THQ value > 1 are indicated in bold.

**Table 6:** Lifetime Carcinogenic Risk (CR) values for Cr and Cd according to different consumption frequencies.

			Cr			Cd		
			5 Day/Week	3 Day/Week	1 Day/Week	5 Day/Week	3 Day/Week	1 Day/Week
*O. melanura*	Spring	A	**4.24** **×** **10^−4^**	**2.54 × 10^−4^**	8.48 × 10^−5^	9.13 × 10^−5^	5.48 × 10^−5^	1.83 × 10^−5^
		C	**9.28 × 10^−4^**	**5.57 × 10^−4^**	**1.86 × 10^−4^**	**2.00 × 10^−4^**	**1.20 × 10^−4^**	4.00 × 10^−5^
	Winter	A	**4.62 × 10^−4^**	**2.77 × 10^−4^**	9.23 × 10^−5^	**1.64 × 10^−4^**	9.85 × 10^−5^	3.28 × 10^−5^
		C	**1.01 × 10^−3^**	**6.06 × 10^−4^**	**2.02 × 10^−4^**	**3.59 × 10^−4^**	**2.15 × 10^−4^**	7.18 × 10^−5^
	Autumn	A	**4.05 × 10^−4^**	**2.43 × 10^−4^**	8.11 × 10^−5^	**3.81 × 10^−4^**	**2.28 × 10^−4^**	7.62 × 10^−5^
		C	**8.87 × 10^−4^**	**5.32 × 10^−4^**	**1.77 × 10^−4^**	**8.30 × 10^−4^**	**5.00 × 10^−4^**	**1.67 × 10^−4^**
	Summer	A	**5.41 × 10^−4^**	**3.25 × 10^−4^**	**1.08 × 10^−4^**	**2.78 × 10^−4^**	**1.67 × 10^−4^**	5.57 × 10^−5^
		C	**1.18 × 10^−3^**	**7.10 × 10^−4^**	**2.37 × 10^−4^**	**6.09 × 10^−4^**	**3.65 × 10^−4^**	**1.22 × 10^−4^**
*S. aurata*	Spring	A	**6.72 × 10^−4^**	**4.03 × 10^−4^**	**1.34 × 10^−4^**	**2.07 × 10^−4^**	**1.24 × 10^−4^**	**4.14 × 10^−5^**
		C	**1.47 × 10^−3^**	**8.81 × 10^−4^**	**2.94 × 10^−4^**	**4.53 × 10^−4^**	**2.72 × 10^−4^**	**9.05 × 10^−5^**
	Winter	A	**5.36 × 10^−4^**	**3.22 × 10^−4^**	**1.07 × 10^−4^**	**1.31 × 10^−4^**	**7.87 × 10^−5^**	**2.62 × 10^−5^**
		C	**1.17 × 10^−3^**	**7.03 × 10^−4^**	**2.34 × 10^−4^**	**2.87 × 10^−4^**	**1.72 × 10^−4^**	**5.74 × 10^−5^**
	Autumn	A	**6.39 × 10^−4^**	**3.83 × 10^−4^**	**1.28 × 10^−4^**	**2.48 × 10^−4^**	**1.49 × 10^−4^**	4.96 × 10^−5^
		C	**1.40 × 10^−3^**	**8.38 × 10^−4^**	**2.79 × 10^−4^**	**5.42 × 10^−4^**	**3.25 × 10^−4^**	**1.08 × 10^−4^**
	Summer	A	**6.14 × 10^−4^**	**3.68 × 10^−4^**	**1.23 × 10^−4^**	**2.05 × 10^−4^**	**1.23 × 10^−4^**	4.11 × 10^−5^
		C	**1.34 × 10^−3^**	**8.06 × 10^−4^**	**2.69 × 10^−4^**	**4.49 × 10^4^**	**2.70 × 10^4^**	8.99 × 10^−5^
*D. dentex*	Spring	A	**7.22 × 10^−4^**	**4.30 × 10^−4^**	**1.44 × 10^−4^**	**1.70 × 10^−4^**	**1.02 × 10^−4^**	3.41 × 10^−5^
		C	**1.58 × 10^−3^**	**9.47 × 10^−4^**	**3.16 × 10^−4^**	**3.73 × 10^−4^**	**2.24 × 10^−4^**	7.46 × 10^−5^
	Winter	A	**1.06 × 10^−3^**	**6.35 × 10^−4^**	**2.12 × 10^−4^**	**1.09 × 10^−4^**	6.52 × 10^−5^	2.17 × 10^−5^
		C	**2.323 × 10^−3^**	**1.393 × 10^−3^**	**4.63 × 10^−4^**	**2.38 × 10^−4^**	**1.43 × 10^−4^**	4.76 × 10^−5^
	Autumn	A	**8.78 × 10^−4^**	**5.27 × 10^−4^**	**1.76 × 10^−4^**	**3.30 × 10^−4^**	**1.98 × 10^−4^**	6.61 × 10^−5^
		C	**1.92 × 10^−3^**	**1.15 × 10^−3^**	**3.84 × 10^−4^**	**7.23 × 10^−4^**	**4.34 × 10^−4^**	**1.45 × 10^−4^**
	Summer	A	**9.99 × 10^−4^**	**6.00 × 10^−4^**	**2.00 × 10^−4^**	**2.19 × 10^−4^**	**1.31 × 10^−4^**	4.38 × 10^−5^
		C	**2.19 × 10^−3^**	**1.31 × 10^−3^**	**4.37 × 10^−4^**	**4.79 × 10^−4^**	**2.88 × 10^−4^**	9.59 × 10^−5^
*P.major*	Spring	A	**6.73 × 10^−4^**	**4.04 × 10^−4^**	**1.35 × 10^−4^**	**1.45 × 10^−4^**	8.72 × 10^−5^	2.91 × 10^−5^
		C	**1.47 × 10^−3^**	**8.84 × 10^−4^**	**2.95 × 10^−4^**	**3.18 × 10^−4^**	**1.91 × 10^−4^**	6.36 × 10^−5^
	Winter	A	**9.12 × 10^−4^**	**5.47 × 10^−4^**	**1.82 × 10^−4^**	**2.84 × 10^−4^**	**1.71 × 10^−4^**	5.69 × 10^−5^
		C	**1.99 × 10^−3^**	**1.20 × 10^−3^**	**3.99 × 10^−4^**	**6.22 × 10^−4^**	**3.73 × 10^−4^**	**1.24 × 10^−4^**
	Autumn	A	**1.00 × 10^−3^**	**6.01 × 10^−4^**	**2.00 × 10^−4^**	**1.63 × 10^−4^**	9.79 × 10^−5^	3.26 × 10^−5^
		C	**2.19 × 10^−3^**	**1.31 × 10^−3^**	**4.38 × 10^−4^**	**3.57 × 10^−4^**	**2.14 × 10^−4^**	7.14 × 10^−5^
	Summer	A	**1.12 × 10^−3^**	**6.70 × 10^−4^**	**2.23 × 10^−4^**	**1.33 × 10^−4^**	8.00 × 10^−5^	2.67 × 10^−5^
		C	**2.44 × 10^−3^**	**1.47 × 10^−3^**	**4.89 × 10^−4^**	**2.92 × 10^−4^**	**1.75 × 10^−4^**	5.83 × 10^−5^
*D. sargus*	Spring	A	**1.01 × 10^−3^**	**6.09 × 10^−4^**	**2.03 × 10^−4^**	4.28 × 10^−5^	2.57 × 10^−5^	8.56 × 10^−6^
		C	**2.22 × 10^−3^**	**1.33 × 10^−3^**	**4.44 × 10^−4^**	9.37 × 10^−5^	5.62 × 10^−5^	1.87 × 10^−5^
	Winter	A	**9.12 × 10^−4^**	**5.47 × 10^−4^**	**1.82 × 10^−4^**	**1.18 × 10^−4^**	7.05 × 10^−5^	2.35 × 10^−5^
		C	**2.00 × 10^−3^**	**1.20 × 10^−3^**	**3.99 × 10^−4^**	**2.57 × 10^−4^**	**1.54 × 10^−4^**	5.14 × 10^−5^
	Autumn	A	**8.46 × 10^−4^**	**5.07 × 10^−4^**	**1.69 × 10^−4^**	**2.33 × 10^−4^**	**1.40 × 10^−4^**	4.66 × 10^−5^
		C	**1.85 × 10^−3^**	**1.11 × 10^−3^**	**3.70 × 10^−4^**	**5.09 × 10^−4^**	**3.06 × 10^−4^**	1.02 × 10^−4^
	Summer	A	**9.78 × 10^−4^**	**5.87 × 10^−4^**	**1.96 × 10^−4^**	**2.25 × 10^−4^**	**1.35 × 10^−4^**	4.50 × 10^−5^
		C	**2.14 × 10^−3^**	**1.28 × 10^−3^**	**4.28 × 10^−4^**	**4.92 × 10^−4^**	**2.95 × 10^−4^**	9.85 × 10^−5^

A, adult; C, children. Exposure time of 5, 3, and 1 days/week. CR values > 10^−5^ are indicated in bold.

## Data Availability

The original contributions presented in this study are included in the article. Further inquiries can be directed to the corresponding author.

## References

[1] Durmus M. (2019). Fish Oil for Human Health: Omega-3 Fatty Acid Profiles of Marine Seafood Species. Food Sci. Technol..

[2] Krittanawong C., Isath A., Hahn J., Wang Z., Narasimhan B., Kaplin S.L., Jneid H., Virani S.S., Tang W.H.W. (2021). Fish Consumption and Cardiovascular Health: A Systematic Review. Am. J. Med..

[3] Stephen N.M., Maradagi T., Kavalappa Y.P., Sharma H., Ponesakki G. (2022). Seafood nutraceuticals: Health Benefits and Functional Properties. Research and Technological Advances in Food Science.

[4] Thomsen S.T., Assunção R., Afonso C., Boué G., Cardoso C., Cubadda F., Garre A., Kruisselbrink J.W., Mantovani A., Pitter J.G. (2022). Human Health Risk–Benefit Assessment of Fish and Other Seafood: A Scoping Review. Crit. Rev. Food Sci. Nutr..

[5] Ayas D., Köşker A.R. (2018). The Effects of Age and Individual Size on Metal Accumulation of *Lagocephalus sceleratus* (Gmelin, 1789) from Mersin Bay, Turkey. Nat. Eng. Sci..

[6] Embaby M.A., Ayesh A.M., Salem S.H., Abdel-Rahman G.N. (2024). Potential Human Health Risk Assessment Associated with Hg, Cd, Pb, and As in Sardines and Shrimp from Four Egyptian Coastal Governorates. Toxicol. Rep..

[7] Lin H., Luo X., Yu D., He C., Cao W., He L., Liang Z., Zhou J., Fang G. (2024). Risk Assessment of As, Cd, Cr, and Pb via the Consumption of Seafood in Haikou. Sci. Rep..

[8] Balali-Mood M., Naseri K., Tahergorabi Z., Khazdair M.R., Sadeghi M. (2021). Toxic mechanisms of five heavy metals: Mercury, lead, chromium, cadmium, and arsenic. Front. Pharmacol..

[9] Younis E.M., Abdel-Warith A.W.A., Al-Asgah N.A., Elthebite S.A., Rahman M.M. (2021). Nutritional value and bioaccumulation of heavy metals in muscle tissues of five commercially important marine fish species from the Red Sea. Saudi J. Biol. Sci..

[10] Banaee M., Zeidi A., Mikušková N., Faggio C. (2024). Assessing metal toxicity on crustaceans in aquatic ecosystems: A comprehensive review. Biol. Trace Elem. Res..

[11] Naccari C., Ferrantelli V., Cammilleri G., Ruga S., Castagna F., Bava R., Palma E. (2024). Trace elements in *Stenella coeruleoalba*: Assessment of marine environmental pollution and dolphin health status. Animals.

[12] Kosker A.R., Gundogdu S., Esatbeyoglu T., Ayas D., Ozogul F. (2023). Metal levels of canned fish sold in Türkiye: Health risk assessment. Front. Nutr..

[13] Karadurmuş U., Köşker A.R., Durmuş M., Aydın M. (2025). Proximate analysis of sea cucumbers (*Parastichopus regalis* Cuvier, 1817) from Türkiye, and health risk assessment of the metal/metalloid contents. J. Food Compos. Anal..

[14] Ejaz A., Ullah S., Ijaz S., Bilal M., Banaee M., Mosotto C., Faggio C. (2024). Bioaccumulation and Health Risk Assessment of Heavy Metals in *Labeo rohita* and *Mystus seenghala* from Jhelum River, Punjab, Pakistan. Water.

[15] Kosker A.R. (2020). Metal and Fatty Acid Levels of Some Commercially Important Marine Species from the Northeastern Mediterranean: Benefits and Health Risk Estimation. Environ. Monit. Assess..

[16] Köşker A.R., Gündoğdu S., Ayas D., Bakan M. (2022). Metal Levels of Processed Ready-To-Eat Stuffed Mussels Sold in Turkey: Health Risk Estimation. J. Food Compos. Anal..

[17] Sirisangarunroj P., Monboonpitak N., Karnpanit W., Sridonpai P., Singhato A., Laitip N., Ornthai N., Yafa C., Judprasong K. (2023). Toxic Heavy Metals and Their Risk Assessment of Exposure in Selected Freshwater and Marine Fish in Thailand. Foods.

[18] Traina A., Bono G., Bonsignore M., Falco F., Giuga M., Quinci E.M., Vitale S., Sprovieri M. (2019). Heavy metals concentrations in some commercially key species from Sicilian coasts (Mediterranean Sea): Potential human health risk estimation. Ecotoxicol. Environ. Saf..

[19] de Almeida Rodrigues P., Ferrari R.G., Kato L.S., Hauser-Davis R.A., Conte-Junior C.A. (2022). A systematic review on metal dynamics and marine toxicity risk assessment using crustaceans as bioindicators. Biol. Trace Elem. Res..

[20] Evangelopoulos A., Karampetsis D., Christidis A., Gubili C., Sapounidis A., Adamidou A., Kamidis N., Koutrakis E. (2024). Non-native fish species in the North Aegean Sea: A review of their distributions integrating unpublished fisheries data. Front. Mar. Sci..

[21] Torcu Koç H., Aka Z., Türker Çakır D. (2005). An Investigation on Fishes of Gökova Bay (Southern Aegean Sea). BAÜ Fen Bil. Enst. Derg..

[22] Almaz A., Or İ., Özbaş B. (2006). Investigation of transit maritime traffic in the strait of Istanbul through simulation modeling and scenario analysis. Int. J. Simul. Syst. Sci. Technol..

[23] Kopasakis K.I., Georgoulas A.N., Angelidis P.B., Kotsovinos N.E. (2012). Numerical modeling of the long-term transport, dispersion, and accumulation of black sea pollutants into the north aegean coastal waters. Estuaries Coasts.

[24] Triantafyllou E., Giamarelou M., Bossioli E., Zarmpas P., Theodosi C., Matsoukas C., Tombrou M., Mihalopoulos N., Biskos G. (2016). Particulate pollution transport episodes from Eurasia to a remote region of northeast Mediterranean. Atmos. Environ..

[25] Basurco B., Lovatelli A., García B. (2011). Current status of Sparidae aquaculture. Sparidae, Biology and Aquaculture of Gilthead Sea Bream and Other Species.

[26] Daban İ.B. (2022). Comparative study on the feeding ecology of the White Seabream, *Diplodus sargus*, and the Black Seabream, *Spondyliosoma cantharus* (Osteichthyes: Sparidae) in the North Aegean Sea. Zool. Middle East.

[27] Canli M., Atli G. (2003). The relationships between heavy metal (Cd, Cr, Cu, Fe, Pb, Zn) levels and the size of six Mediterranean fish species. Environ. Pollut..

[28] US EPA (United States Environmental Protection Agency) (2000). Guidance for Assessing Chemical Contaminant Data for Use in Fish Advisories, Vol. I: Fish Sampling and Analysis.

[29] US EPA (United States Environmental Protection Agency) (2008). Child-Specific Exposure Factors Handbook (Final Report).

[30] (2019). USEPA (United States Environmental Protection Agency). https://www.epa.gov/risk/regional-screening-levels-rsls-equations.

[31] Bat L., Öztekin A., Arıcı E., Fatih Şahin F. (2020). Health risk assessment: Heavy metals in fish from the southern Black Sea. Foods Raw Mater..

[32] Blanchard J., Grosell M. (2005). Effects of salinity on copper accumulation in the common killifish (*Fundulus heteroclitus*). Environ. Toxicol. Chem./SETAC.

[33] Maurya P.K., Malik D.S., Yadav K.K., Kumar A., Kumar S., Kamyab H. (2019). Bioaccumulation and potential sources of heavy metal contamination in fish species in River Ganga basin: Possible human health risks evaluation. Toxicol. Rep..

[34] Khalaf E.M., Taherian M., Almalki S.G., Asban P., Kareem A.K., Alhachami F.R., Almulla A.F., Romero-Parra R.M., Jawhar Z.H., Kiani F. (2024). Relationship between exposure to heavy metals on the increased health risk and carcinogenicity of urinary tract (kidney and bladder). Rev. Environ. Health.

[35] Türkmen M., Türkmen A., Tepe Y. (2008). Metal contaminations in five fish species from Black, Marmara, Aegean and Mediterranean seas, Turkey. J. Chil. Chem. Soc..

[36] Agency for Toxic Substances and Disease Registry (ATSDR) (2012). Toxicological Profile for Vanadium. http://www.atsdr.cdc.gov/ToxProfiles/tp58.pdf.

[37] Capodiferro M., Marco E., Grimalt J.O. (2022). Wild Fish and Seafood Species in the Western Mediterranean Sea with Low Safe Mercury Concentrations. Environ. Pollut..

[38] Tuzen M. (2009). Toxic and essential trace elemental contents in fish species from the Black Sea, Turkey. Food Chem. Toxicol..

[39] Schoofs H., Schmit J., Rink L. (2024). Zinc toxicity: Understanding the limits. Molecules.

[40] Döndü M., Özdemir N., Demirak A., Keskin F., Zeynalova N. (2023). Bioaccumulation and Human Health Risk Assessment of Some Heavy Metals in Sediments, *Sparus aurata* and *Salicornia europaea* in Güllük Lagoon, the South of Aegean Sea. Environ. Sci. Pollut. Res..

[41] Lounas R., Kasmi H., Chernai S., Amarni N., Ghebriout L., Hamdi B. (2021). Heavy metal concentrations in wild and farmed gilthead sea bream from southern Mediterranean Sea—Human health risk assessment. Environ. Sci. Pollut. Res..

[42] Pincemail J., Meziane S. (2022). On the potential role of the antioxidant couple vitamin E/selenium taken by the oral route in skin and hair health. Antioxidants.

[43] Zhang F., Li X., Wei Y. (2023). Selenium and selenoproteins in health. Biomolecules.

[44] Solé M., Omedes S., Rodríguez-Prieto C., Lorenzo M., Casadevall M. (2024). Biomarkers and metal content in white seabream (*Diplodus sargus*) and its relationship with the occurrence of the Abnormal Tough Syndrome. Environ. Pollut. Manag..

[45] European Food Safety Authority (EFSA) (2010). Scientific opinion of the panel on contaminants in the food chain on a request from the European Commission on lead in food. EFSA J..

[46] Loaiza I., Hurtado D., Miglio M., Orrego H., Mendo J. (2015). Tissue-specific Cd and Pb accumulation in Peruvian scallop (*Argopecten purpuratus*) transplanted to a suspended and bottom culture at Sechura Bay, Peru. Mar. Pollut. Bull..

[47] Özyurt G., Tabakoğlu Ş.S., Özyurt C.E. (2021). Metal bioaccumulation in the gill, liver, and muscle of bluefish (*Pomatomus saltatrix*) from the Northeastern Mediterranean and human health risk assessment associated with their seasonal consumption. Arch. Environ. Contam. Toxicol..

[48] Yu X., Khan S., Khan A., Tang Y., Nunes L.M., Yan J., Ye X., Li G. (2020). Methyl mercury concentrations in seafood collected from Zhoushan Islands, Zhejiang, China, and their potential health risk for the fishing community: Capsule: Methyl mercury in seafood causes potential health risk. Environ. Int..

[49] Külcü A.M., Ayas D., Köşker A.R., Yatkin K. (2014). The Investigation of metal and mineral levels of some marine species from the Northeastern Mediterranean Sea. J. Mar. Biol. Oceanogr..

[50] Turanlı N., Gedik K. (2021). Spatial trace element bioaccumulation along with consumer risk simulations of Mediterranean mussels in coastal waters of Turkey. Environ. Sci. Pollut. Res..

[51] Garai P., Banerjee P., Mondal P., Saha N.C. (2021). Effect of heavy metals on fishes: Toxicity and bioaccumulation. J Clin Toxicol. S.

[52] Santhosh K., Kamala K., Ramasamy P., Musthafa M.S., Almujri S.S., Asdaq S.M.B., Sivaperumal P. (2024). Unveiling the silent threat: Heavy metal toxicity devastating impact on aquatic organisms and DNA damage. Mar. Pollut. Bull..

[53] Tokatlı C., Emiroğlu Ö., Çiçek A., Köse E., Bașkurt S., Aksu S., Uğurluoğlu A., Șahİn M., Baștatlı Y. (2016). Investigation of Toxic Metal Bioaccumulations in Fishes of Merİç River Delta (Edirne). Anadolu Univ. J. Sci. Technol. C. Life Sci. Biotechnol..

[54] Varol M., Kaçar E. (2023). Bioaccumulation of metals in various tissues of fish species in relation to fish size and gender and health risk assessment. Curr. Pollut. Rep..

[55] Kır İ., Özan S.T., Tuncay Y. (2007). The seasonal variations of some heavy metals in Kovada Lake’s water and sediment. Ege J. Fish. Aquat. Sci..

[56] Özmen H., Külahçı F., Çukurovalı A., Doğru M. (2004). Concentrations of heavy metal and radioactivity in surface water and sediment of Hazar Lake (Elazığ, Turkey). Chemosphere.

[57] Kalay M., Koyuncu C.E., Dönmez A.E. (2004). Mersin körfezi’nden yakalanan *Sparus aurata* (L. 1758) ve *Mullus barbatus* (L. 1758)’un kas ve karaciğer dokularındaki kadmiyum düzeylerinin karşılaştırılması. Ekoloji.

[58] Förstner U., Wittmann G.T., FAO/WHO (1983). Evaluation of Certain Food Additives and Contaminants: Twenty-Seventh Report of the Joint FAO Expert Committee on Food Additives.

[59] JECFA (2010). Summary and Conclusions by the JECFA.

[60] FAO/WHO (2019). General Standard for Contaminants and Toxins in Food and Feed. CODEX Aliment..

[61] JECFA (2011). Evaluation of certain food additives and contaminants. Seventyfourth Report of the Joint FAO/WHO Expert Committee on Food Additives.

[62] EFSA Panel on Contaminants in the Food Chain (CONTAM) (2009). Scientific opinion on arsenic in food. EFSA J..

[63] EFSA Panel on Biological Hazards (BIOHAZ) (2014). Statement on the update of the list of QPS-recommended biological agents intentionally added to food or feed as notified to EFSA 1: Suitability of taxonomic units notified to EFSA until October 2014. EFSA J..

[64] Storelli M.M., Cuttone G., Marcotrigiano G.O. (2011). Distribution of trace elements in the tissues of smooth hound *Mustelus mustelus* (Linnaeus, 1758) from the southern–eastern waters of Mediterranean Sea (Italy). Environ. Monit. Assess..

[65] Mol S., Kahraman A.E., Ulusoy S. (2018). Potential health risks of heavy metals to the Turkish and Greek populations via consumption of Spiny Dogfish and Thornback Ray from the Sea of Marmara. Turk. J. Fish. Aquat. Sci..

[66] Korkmaz C., Aya Ö., Ersoysal Y., Köroğlu M.A., Erdem C. (2019). Heavy metal levels in muscle tissues of some fish species caught from northeast Mediterranean: Evaluation of their effects on human health. J. Food Compos. Anal..

[67] Uçar Y. (2020). Elemental Compositions and Fatty Acid Profiles of Bogue Fish (*Boops boops*) from Mediterranean Coast: A Comprehensive Evaluation of the Potential Effects on Human Health. Biol. Trace Elem. Res..

